# Insecticide resistance monitoring in field populations of *Chilo suppressalis* Walker (Lepidoptera: Crambidae) from central China

**DOI:** 10.3389/fphys.2022.1029319

**Published:** 2022-11-01

**Authors:** Haoran Meng, Rong Huang, Hu Wan, Jianhong Li, Junkai Li, Xiaolei Zhang

**Affiliations:** ^1^ Hubei Engineering Technology Center of Forewarning and Management of Agricultural and Forestry Pests, College of Agriculture, Yangtze University, Jingzhou, China; ^2^ Hubei Insect Resources Utilization and Sustainable Pest Management Key Laboratory, College of Plant Science and Technology, Huazhong Agricultural University, Wuhan, China

**Keywords:** *Chilo suppressalis* Walker, triazophos, chlorpyrifos, abamectin, insecticide resistance monitoring

## Abstract

*Chilo suppressalis* Walker (Lepidoptera: Crambidae) is a devastating rice crop pest in China. Chemical insecticides have been used to effectively managing *C*. *suppressalis* field populations in most of China’s agricultural regions. However, due to the intensive and extensive application of these insecticides, *C*. *suppressalis* has developed widespread resistance to many active ingredients. Thus, insecticide resistance development is a genuine concern for all crop specialists and growers. In this study, using the topical application method, we assessed the susceptibility of forty-six field populations of *C*. *suppressalis* to three insecticides in three Central Chinese provinces from 2010 to 2021. Our findings revealed that field populations of *C*. *suppressalis* built moderate to high levels of resistance to triazophos (Resistance Ratio (RR) = 41.9–250.0 folds), low to moderate levels of resistance to chlorpyrifos (RR = 9.5–95.2 folds), with the exception of the Zhijiang population in 2013 and the Xinyang population in 2015 at 4.8 folds and 3.4 folds resistance rates, respectively, despite showing susceptibility, and low and moderate levels of resistance to abamectin (RR = 4.1–53.5 folds). There were significant correlations between the activity of the detoxification enzymes (CarE) and the log LD_50_ values of triazophos. These results should help effective insecticide resistance management strategies reduce the risk of rapid build-ups of resistance to insecticides and slow down the process of selection for insecticide resistance.

## Introduction

The striped rice borer, *Chilo suppressalis* Walker (Lepidoptera: Crambidae), is a devastating rice pest in Asia, Oceania and south Europe (Meng et al., 2019). The larvae of *C. suppressails* bore into the leaf sheaths and stems of rice, then feed on the tissues of leaf sheath and stem of rice, forming dead sheaths, dead hearts and white heads (Lu et al., 2017). Since the 1990s, *C*. *suppressalis* populations have spread out and caused severe damages to rice-growing areas in China due to the use of hybrid varieties and changes in the rice cultivation system (Zhu and Cheng, 2013; Xu et al., 2015). *C*. *suppressalis* outbreaks have led to reduced yields of rice and related economic losses (Li et al., 2020), with statistics demonstrating that they have damaged more than 14 million hectares of rice fields in China every year for the last 10 years by the National Agro-Tech Extension and Service Center (NATESC) (NATESC, 2022).

To fight the devastation caused by these insects, farmers have adopted the widespread application of insecticides to manage *C*. *suppressalis* over the last several decades (Zhao, 2019). Unfortunately, this has resulted in the evolution of insecticide resistance by field populations of *C*. *suppressalis*. *C*. *suppressalis* currently has different levels of resistance to organochlorines, organophosphates, carbamates, phenylpyrazoles, avermectins, nereistoxin analogues and diamide groups in China (Huang et al., 2017; Lu et al., 2017; Yao et al., 2017; Xu et al., 2018; Mao et al., 2019; Wei et al., 2019; Zhao, 2019; Huang et al., 2020; Sun et al., 2021; Zhao et al., 2021). The evolution of *C*. *suppressalis’* resistance to insecticides is well documented in the Arthropod Pesticide Resistance Database (APRD) (Mota-Sanchez and Wise, 2022). The development of insecticide resistance by *C*. *suppressalis* has diminished the ability to control field populations of this insecticide. Nonetheless, this resistance has only served to increase the use of insecticides to control *C*. *suppressalis*.

Chlorpyrifos, triazophos, and abamectin are the major insecticides to have been predominantly used to control *C*. *suppressalis* for several decades (Zhao, 2019). In China, triazophos was used to control *C*. *suppressalis* in the early 1990s, chlorpyrifos was adopted as an alternative agent in rice fields after the field population of *C*. *suppressalis* developed high resistance levels to triazophos, and abamectin has been in use against field populations of *C*. *suppressalis* since the end of the 20th century (Yao, 2015). Presently, only a limited number of insecticides can contain *C*. *suppressalis* in China. Hence, monitoring and understanding the status of insecticide resistance is fundamental to successful resistance management (Sun et al., 2021; Xu et al., 2021). In addition, insecticide resistance levels and the speed of development of resistance by field populations of *C*. *suppressalis* differ between regions depending on the closeness of the association with the cultivation system, the history of insecticide use, and the degree of insecticide use in a country or region. Therefore, monitoring insecticide resistance by *C*. *suppressalis* field populations in different areas is of great significance to the rational choice of insecticides, the rotation of insecticide MoA (modes of action) groups, insecticide mixtures, reducing the risk of insecticide resistance, preventing further insecticide resistance development, and improving insecticide control efficiency.

Managing insecticide resistance requires knowing the mechanism of insecticide resistance (Zhang et al., 2017a). Previous studies have demonstrated how understanding the mechanism behind the resistance to insecticides is critical to pest management (He et al., 2012; Zhang et al., 2017b). Insecticide resistance often stems from gene regulatory changes that culminate in increased detoxification activities, such as carboxylesterase (CarE), glutathione *S*-transferase (GST), and cytochrome P450 monooxygenases (P450) (Heckel, 2012; Mao et al., 2019; Bhatt et al., 2021; Meng et al., 2022; Nauen et al., 2022). The increase in these detoxifying enzymes is the most common resistance mechanism (Heckel, 2012; Enders et al., 2020). Furthermore, insecticides with cross-resistance share the same resistance mechanisms, such as alternate target-sites, enhanced enzyme systems, or reduced penetration (Qian et al., 2008). Thus, applying only one of them could still result in the resistance to all of them, and these insecticides cannot be used rotationally in resistance management.

The present study monitored insecticide resistance by field populations of *C*. *suppressalis* to organophosphate insecticides (chlorpyrifos and triazophos) and an avermectin insecticide (abamectin) from 2010 to 2021 to evaluate the levels of resistance to insecticides by these field populations. The outcome of this investigation could provide a scientific basis for the rational selection of insecticides and delay the development of resistance by *C*. *suppressalis* field populations.

## Materials and methods

### Insects

Forty-six field populations of *C*. *suppressalis* were gathered from the rice paddy fields of Gongan, Jianli, Wuxue, Zaoyang, Zhijiang, Ezhou, Xiaogan, Chibi, Xiantao, Qianjiang, Songzi, Changde, Changsha, and Xinyang in Central China (Table 1) from 2010 to 2021. More than 100–2000 larvae were collected from each site. The fourth instar larvae were used for bioassays. The fourth-instar larvae of the first (F1) and second (F2) generations were used for the susceptibility bioassay.

**TABLE 1:** Sampling sites, dates, and developmental stages of *C*. *suppressalis* collected from fields.

Population	Location	Collection date (year-month-day)	Site	Insect stage
WX-2010	Wuxue, Hubei	2010-06-23	30.11° N, 115.59° E	larva
WX-2011	Wuxue, Hubei	2011-06-30	30.11° N, 115.59° E	larva
WX-2012	Wuxue, Hubei	2012-07-01	30.11° N, 115.59° E	larva
WX-2013	Wuxue, Hubei	2013-07-02	30.11° N, 115.59° E	larva
WX-2014	Wuxue, Hubei	2014-07-02	30.11° N, 115.59° E	larva
WX-2015	Wuxue, Hubei	2015-06-02	30.11° N, 115.59° E	larva
ZJ-2010	Zhijiang, Hubei	2010-07-05	30.26° N, 111.55° E	larva
ZJ-2011	Zhijiang, Hubei	2011-06-24	30.26° N, 111.55° E	larva
ZJ-2012	Zhijiang, Hubei	2012-06-23	30.26° N, 111.55° E	larva
ZJ-2013	Zhijiang, Hubei	2013-06-29	30.26° N, 111.55° E	larva
ZJ-2014	Zhijiang, Hubei	2014-07-04	30.26° N, 111.55° E	larva
ZJ-2015	Zhijiang, Hubei	2015-07-07	30.26° N, 111.55° E	larva
JL-2010	Jianli, Hubei	2020-07-28	29.91° N, 112.77° E	larva
JL-2011	Jianli, Hubei	2011-06-25	29.91° N, 112.77° E	larva
JL-2012	Jianli, Hubei	2012-06-30	29.91° N, 112.77° E	larva
JL-2013	Jianli, Hubei	2013-06-29	29.91° N, 112.77° E	larva
JL-2014	Jianli, Hubei	2014-06-25	29.91° N, 112.77° E	larva
ZY-2010	Zaoyang, Hubei	2010-07-30	31.98° N, 112.76° E	larva
ZY-2011	Zaoyang, Hubei	2011-08-01	31.98° N, 112.76° E	larva
ZY-2012	Zaoyang, Hubei	2012-08-07	31.98° N, 112.76° E	larva
ZY-2013	Zaoyang, Hubei	2013-08-12	31.98° N, 112.76° E	larva
ZY-2014	Zaoyang, Hubei	2014-08-03	31.98° N, 112.76° E	larva
ZY-2015	Zaoyang, Hubei	2015-07-23	31.98° N, 112.76° E	larva
GA-2010	Gongan, Hubei	2010-08-01	30.05° N, 112.19° E	larva
GA-2011	Gongan, Hubei	2011-07-12	30.05° N, 112.19° E	larva
GA-2012	Gongan, Hubei	2012-07-22	30.05° N, 112.19° E	larva
GA-2013	Gongan, Hubei	2013-06-30	30.05° N, 112.19° E	larva
GA-2014	Gongan, Hubei	2014-07-23	30.05° N, 112.19° E	larva
GA-2015	Gongan, Hubei	2015-07-03	30.05° N, 112.19° E	larva
EZ-2010	Ezhou, Hubei	2010-07-20	30.35° N, 114.71° E	larva
EZ-2011	Ezhou, Hubei	2011-07-14	30.35° N, 114.71° E	larva
EZ-2012	Ezhou, Hubei	2012-06-20	30.35° N, 114.71° E	larva
EZ-2013	Ezhou, Hubei	2013-06-20	30.35° N, 114.71° E	larva
TM-2015	Tianmen, Hubei	2015-06-17	30.42° N, 114.46° E	larva
XG-2015	Xiaogan, Hubei	2015-06-03	30.95° N, 114.07° E	larva
CB-2015	Chibi, Hubei	2015-06-27	29.66° N, 113.85° E	larva
CS-2015	Changsha, Hunan	2015-06-15	28.18° N, 112.57° E	larva
XY-2015	Xinyang, Henan	2015-06-12	32.25° N, 113.88° E	larva
QJ-2020	Qianjiang, Hubei	2020-08-25	30.44° N, 112.98° E	larva
QJ-2021	Qianjiang, Hubei	2021-08-16	30.39° N, 112.66° E	larva
SZ-2020	Songzi, Hubei	2020-06-12	30.01° N, 111.90° E	larva
SZ-2021	Songzi, Hubei	2021-08-10	30.01° N, 111.90° E	larva
XT-2020	Xiantao, Hubei	2020-07-01	30.39° N, 113.17° E	larva
XT-2021	Xiantao, Hubei	2021-06-29	30.37° N, 113.35° E	larva
CD-2020	Changde, Hunan	2020-08-16	29.62° N, 111.78° E	larva
CD-2021	Changde, Hunan	2021-06-22	29.63° N, 111.74° E	larva

### Chemicals

Technical grade chlorpyrifos (98%) (CAS#: 2921-88-2) and triazophos (80%) (CAS#: 24017-47-8) were acquired from Hubei Kangbaotai Fine-Chemicals CO., Ltd., Hubei, China, and technical grade abamectin (97%) (CAS#: 71751-41-2) was procured from Chemtac Chemical CO., Ltd., Hebei, China. 1-naphtol (SCR®) (CAS#: 90-15-3), Coomassie brilliant blue G250 (Our-chem®) (CAS#: 6101-58-1), 1-phenyl-2-thiourea (Ourchem®) (CAS#: 103-85-5), 4-Nitroanisole (Ourchem®) (CAS#: 100-17-4) and Fast blue B salt (Ourchem®) (CAS#: 14263-94-6) were procured from Sinopharm Chemical Reagent Co., Ltd., Shanghai, China. 1-naphthyl acetate (CAS#: 830-81-9) and phenylmethanesulfonyl fluoride (PMSF) (CAS#: 329-98-6) were procured from Shanghai Macklin Biochemical Co. Ltd., Shanghai, China. Dithiothreitol (DTT) (CAS#: 3483-12-3) was procured from Beijing Solarbio Science & Technology Co., Ltd., Beijing, China.

### Bioassays

Insecticide resistance to chlorpyrifos, triazophos and abamectin by *C*. *suppressalis* was assessed using topical application bioassays (He et al., 2007; Su et al., 2014a). All the insecticides were dissolved in acetone and then diluted into a series of acetone concentrations. Five to six doses of each insecticide were created, with each dose (concentration) made in triplicates. The control experiment was treated with acetone in place of an insecticide solution. Four filter papers were placed on the base of each petri dish (9 cm diameter) and hydrated by pipetting 5 ml of water onto the filter papers. Ten larvae were transferred onto rice stems (approximately 5–7 cm sections of the stem) in petri dishes (9 cm in diameter) for treatment with each replicate of a dose. The culture conditions for the treated larvae were controlled at 28 ± 1°C and a photoperiod of 16:8 h (L:D). Mortalities were checked 48 h later to determine the impact of chlorpyrifos and triazophos, and 72 h later for abamectin effect.

### Enzyme assays

Detoxification enzyme sources were isolated from the third instar larvae of *C*. *suppressalis via* the following method. The 4^th^ instar larvae were placed in a 2.0 ml homogenizer, inundated with 1 ml 0.1 mol/L phosphate buffer (at 4°C and pH 7.5), and homogenized in an ice-bath. The resulting solutions were collected and centrifuged at 18,000 r/min at 4°C for 30 min, and the obtained supernatants were used as detoxification enzyme solutions for enzyme assays. Protein content was determined utilizing the protein assay kit (Bio-Rad Laboratories, Inc., Hercules, CA, United States), employing bovine serum albumin as the standard. Enzymatic assays were conducted in three repetitions, and each assay was repeated at least twice.

Carboxylesterase (CarEs) activity was measured as demonstrated previously (Zhang et al., 2017a) but with a slight modification. 1 ml of the substrate solution of naphthyl acetate (1 × 10^−6^ mol/L physostigmine) was introduced into an EP-tube, preheated in a water bath at 37°C for 2 min, and doused with 0.20 ml of a diluted enzyme source. The mixture was then left to react at 37°C for 15 min, after which approximately 0.2 ml of the colorimetric reagent FAST Blue B was applied to terminate the reaction. Absorbance was measured with a microplate reader (Bio-Rad) at 600 nm after 30 min of incubation at room temperature.

Glutathione *S*-transferase activity was assessed using 1-chloro-2, 4-dinitrobenzene (CDNB) as a substrate as described previously (Mao et al., 2019). 50 μl of an enzyme solution was added to a mixture of 790 μl of phosphate buffer (pH 6.5), 30 μl of a substrate (30 mM CDNB), and 30 μl of reduced glutathione (50 mM GSH), and the change in absorbance was measured at 340 nm at 5 s intervals for 2 min.

Cytochrome P450 monooxygenase activity was evaluated using p-nitroanisole (PNA) as the substrate as established previously (Zhang et al., 2017a). 675 μl of an enzyme source was added to a mixture of 750 μl of 2 μm PNA (p-nitroanisole) and 75 μl of 9.6 μM NADPH, and the change in absorbance was measured at 405 nm after 30 min of incubation at 34°C.

### Statistical analysis

Mortality data were corrected using Abbott’s formula, and LD_50_ values and 95% confidence interval (CI) were calculated employing the probit analysis. The resistance ratio (RR) was determined by dividing the LD_50_ value of a field population by the corresponding LD_50_ value of the susceptible baseline (Table 2). The degree of resistance was classified as demonstrated by Shao et al. (2013): resistance with RR ≤ 5 folds was classified as susceptibility, RR = 5−10 folds as a low resistance level, RR = 10−100 folds as a moderate resistance level and RR > 100 folds as a high resistance level. Correlations between variables were established using the Pearson method *via* the IBM SPSS Statistics 25 software package. *p* < 0.05 was considered statistically significant.

**TABLE 2:** The LD_50_ values of the susceptibility baseline of *C*. *suppressalis.*

Insecticide group	Insecticide	LD_50_ (95% CI^a^) μg/larva	Reference
Organophosphates	Chlorpyrifos	0.0084 (0.0073–0.0095)	Su et al. (2014a)
	Triazophos	0.0062 (0.0051–0.0074)	He et al. (2007)
Avermectins	Abamectin	0.00017 (0.00014–0.00020)	He et al. (2007)

^a^ CI, confidence limit.

## Results

### Insecticide resistance

**FIGURE 1:**
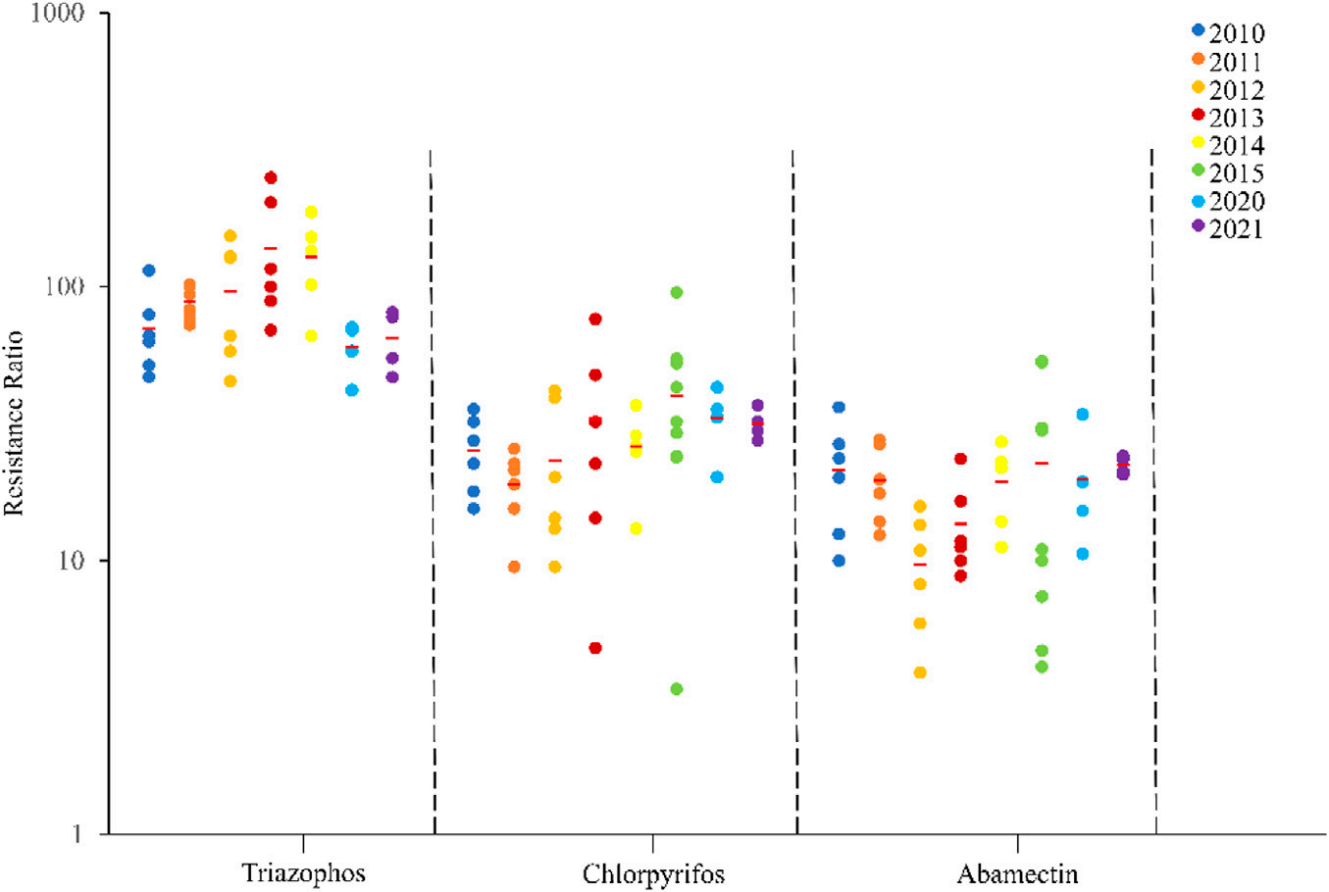
Resistance levels of *C. suppressalis* field populations collected in Centra China from 2010-2021 to 3 insecticides. The circle dots represent the resistance ratios of different populations of *C. suppressalis* to different insecticides. Red horizontal lines across the scatter diagram represent the mean values of the resistance ratios of the different populations.

**FIGURE 2:**
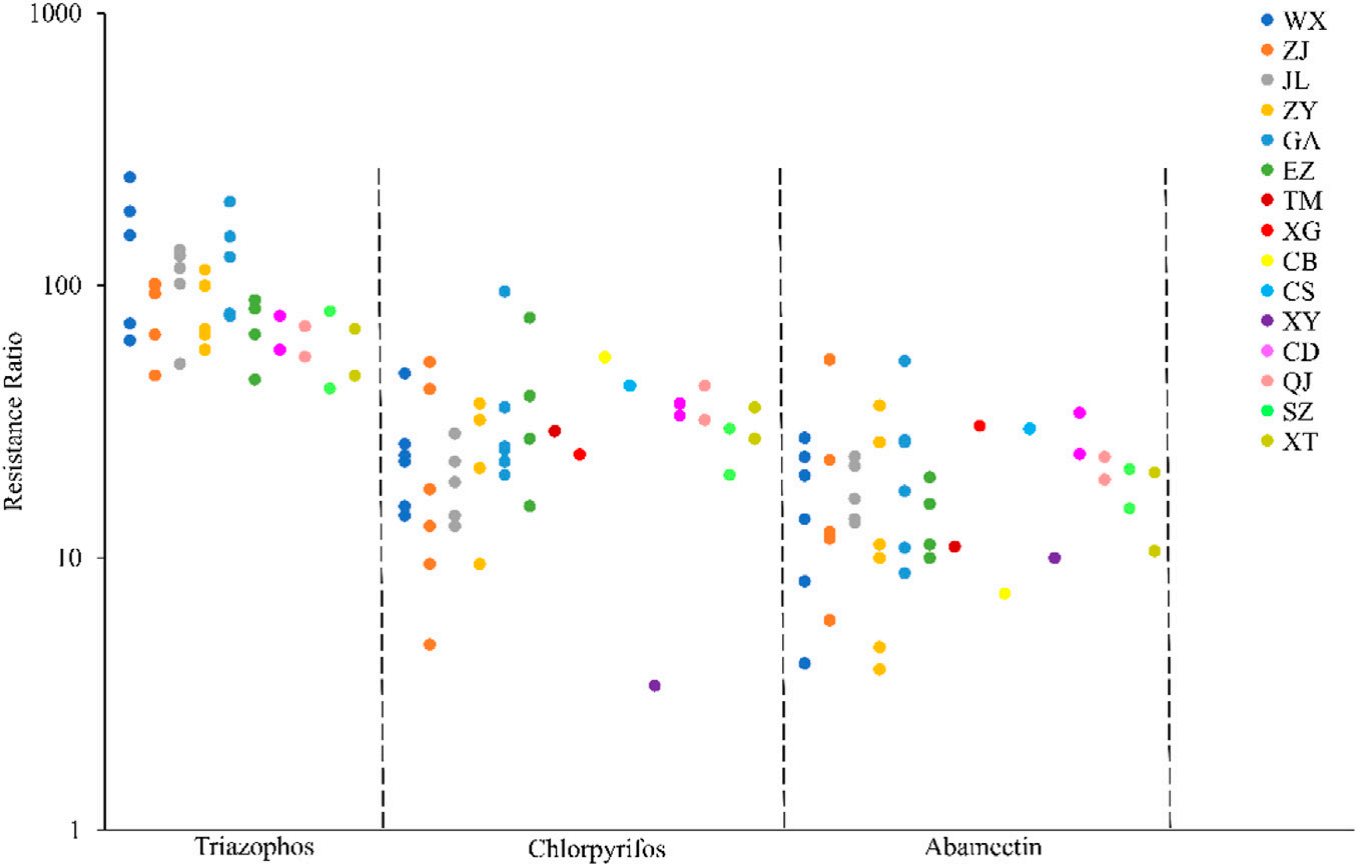
Comparison of the resistance levels of *C. suppressalis* field populations collected in Centra China to 3 insecticides. The circle dots represent the resistance ratios of different populations of *C. suppressalis* to different insecticides.

The field populations of *C*. *suppressalis* developed low to moderate levels of resistance to triazophos (Table 3; Figure 1, Figure 2). Specifically, from 2010 to 2014, 41.4% of the field populations of *C*. *suppressalis* were highly resistant to triazophos (RR = 101.6−250.0 folds), while 58.6% were moderately resistant (RR = 45.2−100.0 folds) (Table 3). In general, the field populations of *C*. *suppressalis* showed moderate levels of resistance from 2020 to 2021 (RR = 41.9−80.6 folds) (Table 3). There were also high levels of resistance to triazophos in 2010 by the field populations of *C*. *suppressalis* collected from Zaoyang, the first population to highly resist the effects of triazophos in Hubei. The LD_50_ values ranged from 0.11 to 1.55 µg/larva, with a 14.1 folds variation, showing relatively inhomogeneous responses across the field populations. The results revealed that *C. suppressalis’* resistance to triazophos increased first and then decreased in the last decade (Figure 1). However, *C*. *suppressalis’* resistance ratio to triazophos in the same location fluctuated greatly across the years (Figure 2).

**TABLE 3:** The resistance levels of *C*. *suppressalis* field populations to insecticides.

Population	Triazophos		Chlorpyrifos		Abamectin	
	LD_50_ (95%CI^a^) μg/larva	RR^b^	LD_50_ (95%CI) μg/larva	RR	LD_50_ (95%CI) μg/larva	RR
WX-2010	0.39 (0.28–0.54)	62.9	0.13 (0.10–0.18)	15.5	0.0034 (0.0026–0.0048)	20.1
WX-2011	0.45 (0.26–0.78)	72.6	0.19 (0.10–0.78)	22.6	0.0047 (0.0035–0.0065)	27.6
WX-2012	0.95 (0.72–1.25)	153.2	0.12 (0.08–0.21)	14.3	0.0014 (0.0010–0.0020)	8.2
WX-2013	1.55 (0.94–2.58)	250.0	0.40 (0.15–0.85)	47.6	0.0040 (0.0022–0.0074)	23.5
WX-2014	1.16 (0.76–2.58)	187.1	0.22 (0.10–0.99)	26.2	0.0022 (0.0014–0.0034)	13.9
WX-2015	nt		0.20 (0.13–0.30)	23.8	0.0007 (0.0002–0.0025)	4.1	
ZJ-2010	0.29 (0.23–0.37)	46.8	0.15 (0.11–0.21)	17.9	0.0021 (0.0014–0.0030)	12.5
ZJ-2011	0.58 (0.43–0.79)	93.6	0.08 (0.05–0.14)	9.5	0.0021 (0.0013–0.0030)	12.4
ZJ-2012	0.41 (0.24–0.70)	66.1	0.35 (0.26–0.46)	41.7	0.0010 (0.0009–0.0012)	5.9
ZJ-2013	0.62 (0.56–0.68)	100.0	0.04 (0.02–0.08)	4.8	0.0020 (0.0011–0.0033)	11.8
ZJ-2014	0.63 (0.40–1.56)	101.6	0.11 (0.05–0.41)	13.1	0.0039 (0.0025–0.0069)	22.9
ZJ-2015	nt		0.44 (0.24–0.83)	52.4	0.0091 (0.0072–0.0116)	53.5	
JL-2010	0.32 (0.23–0.44)	51.6	0.19 (0.13–0.29)	22.6	0.0040 (0.0030–0.0060)	23.6
JL-2011	0.63 (0.42–0.94)	101.6	0.16 (0.09–0.47)	19.0	0.0024 (0.0017–0.0030)	13.9
JL-2012	0.80 (0.60–1.08)	129.0	0.11 (0.08–0.20)	13.1	0.0023 (0.0015–0.0043)	13.5
JL-2013	0.72 (0.57–0.92)	116.1	0.12 (0.07–0.25)	14.3	0.0028 (0.0017–0.0042)	16.5
JL-2014	0.84 (0.55–1.73)	135.5	0.24 (0.13–0.84)	28.6	0.0037 (0.0021–0.0078)	21.8
ZY-2010	0.71 (0.52–0.96)	114.5	0.27 (0.19–0.55)	32.1	0.0062 (0.0041–0.0126)	36.3
ZY-2011	0.62 (0.54–0.72)	100.0	0.18 (0.11–0.43)	21.4	0.0045 (0.0035–0.0060)	26.6
ZY-2012	0.36 (0.26–0.49)	58.1	0.08 (0.06–0.14)	9.5	0.0007 (0.0004–0.0010)	3.9
ZY-2013	0.43 (0.28–0.65)	69.4	0.27 (0.13–1.06)	32.1	0.0017 (0.0009–0.0026)	10.0
ZY-2014	0.41 (0.27–0.84)	66.1	0.31 (0.15–1.71)	36.9	0.0019 (0.0012–0.0031)	11.2
ZY-2015	nt		0.27 (0.19–0.40)	32.1	0.0008 (0.0007–0.0011)	4.7	
GA-2010	0.49 (0.40–0.60)	79.0	0.30 (0.18–1.02)	35.7	0.0045 (0.0033–0.0073)	26.6
GA-2011	0.48 (0.37–0.62)	77.4	0.21 (0.12–0.56)	25.6	0.0030 (0.0023–0.0038)	17.6
GA-2012	0.79 (0.63–0.99)	127.4	0.17 (0.11–0.40)	20.2	0.0019 (0.0014–0.0029)	10.9
GA-2013	1.26 (0.79–2.01)	203.2	0.19 (0.10–0.65)	22.6	0.0015 (0.0008–0.0025)	8.8
GA-2014	0.94 (0.58–2.14)	151.6	0.21 (0.12–0.62)	25.0	0.0046 (0.0030–0.0090)	27.1
GA-2015	nt		0.80 (0.33–1.95)	95.2	0.0090 (0.0041–0.0200)	52.9	
EZ-2010	0.41 (0.27–0.67)	66.2	0.23 (0.16–0.40)	27.4	0.0017 (0.0011–0.0024)	10.0
EZ-2011	0.51 (0.42–0.62)	82.3	0.13 (0.09–0.25)	15.5	0.0034 (0.0026–0.0044)	19.8
EZ-2012	0.28 (0.25–0.30)	45.2	0.33 (0.27–0.41)	39.3	0.0027 (0.0021–0.0034)	15.8
EZ-2013	0.55 (0.35–0.85)	88.7	0.64 (0.55–0.74)	76.2	0.0019 (0.0015–0.0023)	11.2
TM-2015	nt		0.25 (0.19–0.32)	29.2	0.0019 (0.0013–0.0024)	11.0	
XG-2015	nt		0.20 (0.13–0.35)	24.0	0.0052 (0.0034–0.0080)	30.5	
CB-2015	nt		0.46 (0.35–0.66)	54.6	0.0013 (0.0010–0.0016)	7.4	
CS-2015	nt		0.36 (0.24–0.54)	42.9	0.0051 (0.0029–0.012)	29.8	
XY-2015	nt		0.03 (0.02–0.04)	3.4	0.0017 (0.0016–0.0018)	10.0	
CD-2020	0.36 (0.26–0.51)	58.1	0.28 (0.18–0.39)	33.3	0.0058 (0.0042–0.0083)	34.1
CD-2021	0.48 (0.29–0.69)	77.4	0.31 (0.23–0.48)	36.9	0.0041 (0.0029–0.0064)	24.1
QJ-2020	0.44 (0.39–0.93)	71.0	0.36 (0.26–0.51)	42.9	0.0033 (0.0024–0.0044)	19.4
QJ-2021	0.34 (0.25–0.48)	54.8	0.27 (0.20–0.37)	32.1	0.0040 (0.0030–0.0057)	23.5
SZ-2020	0.26 (0.19–0.38)	41.9	0.17 (0.12–0.24)	20.2	0.0026 (0.0018–0.0038)	15.2
SZ-2021	0.50 (0.35–0.71)	80.6	0.25 (0.18–0.34)	29.8	0.0036 (0.0021–0.0052)	21.2
XT-2020	0.43 (0.32–0.63)	69.4	0.30 (0.21–0.40)	35.7	0.0018 (0.0014–0.0024)	10.6
XT-2021	0.29 (0.20–0.39)	46.7	0.23 (0.15–0.31)	27.4	0.0035 (0.0026–0.0049)	20.6

Nt, not test.

^a^ CI, confidence limit.

^b^ RR, resistance ratio was calculated by dividing the LD_50_ value of a field population by the corresponding LD_50_ value of the susceptibility baseline of *C*. *suppressalis*.

The field populations of *C*. *suppressalis* showed susceptibility and low to moderate levels of resistance to chlorpyrifos (Table 3; Figure 1, Figure 2). Forty-two of forty-six field populations of *C*. *suppressalis* maintained moderate resistance levels to chlorpyrifos from 2010 to 2021 (Table 3), with the other four collected from Zhijiang in 2011 and 2013, Zaoyang in 2012 and Xinyang in 2015 displaying low levels of resistance to chlorpyrifos (Table 3). The LD_50_ values ranged from 0.04 to 0.64 µg/larva, with a 16.0 folds variation, pointing to relatively inhomogeneous responses among the field populations. Meanwhile, there were variations in the levels of resistance to chlorpyrifos within the same regions of Centra China across the years (Figure 2). However, the tendency for the degree of resistance to chlorpyrifos to rise was not notable (Figure 1).

The field populations of *C*. *suppressalis* exhibited susceptibility, as well as low and moderate levels of resistance to abamectin (Table 3; Figure 1, Figure 2). Twenty-eight of thirty-one field populations of *C*. *suppressalis* gathered in 2010, 2011, 2013, 2014, 2020, and 2021 built moderate levels of resistance (RR = 11.2−36.3 folds) to abamectin, with the other three populations (Ezhou population in 2010 and Zaoyang and Gongan populations in 2013) displaying low levels of resistance (8.8−10 folds) against abamectin (Table 3). According to analyses of the 2012 impact, field populations from Jianli, Ezhou, and Gongan were moderately resistant to abamectin, and the remaining populations remained lowly resistant (Table 3). Six of nine field populations amassed from Central China in 2015 demonstrated moderate levels of resistance to abamectin, with the other three from Wuxue, Zaoyang and Chibi maintaining low levels of resistance (Table 3). Like *C. suppressalis’* resistance to chlorpyrifos, the tendency for the degree of resistance to abamectin to rise was not apparent; however, the resistance ratio to abamectin varied significantly within the same regions across the years (Figures 1, 2).

### Enzyme activity

**FIGURE 3:**
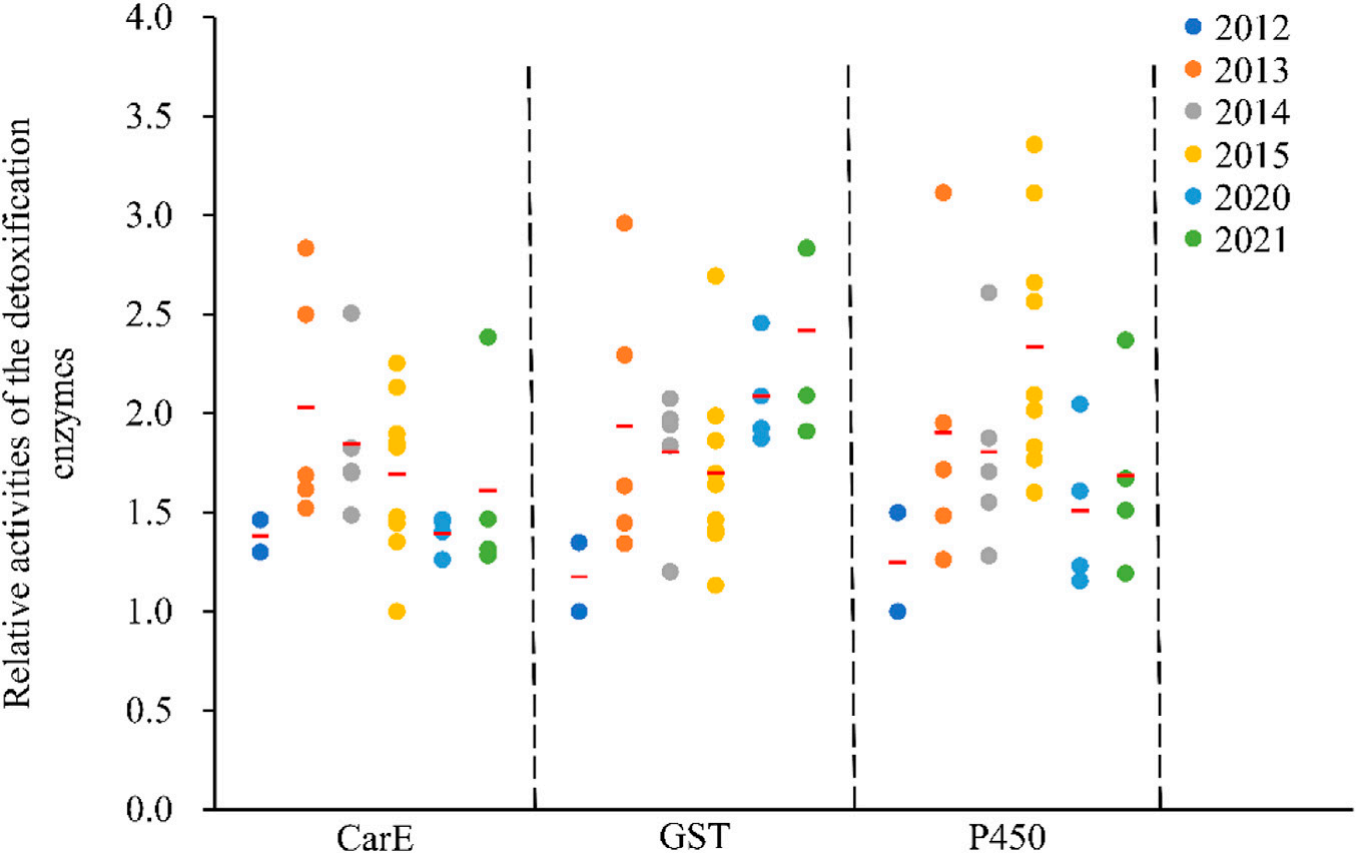
Relative activities of the detoxification enzymes in the field populations of *C. suppressalis* from 2012-2021. The relative activity of a detoxification enzyme corresponds to the ratio of the said detoxification enzyme activity and the minimum enzyme activity of the same enzyme. Red horizontal lines across the scatter diagram represent the mean values of the activities of the detoxification enzymes of the different populations.

Enzyme activity, including carboxylesterase (CarE), glutathione *S*-transferase (GST) and cytochrome P450 monooxygenase (P450) differed between *C*. *suppressalis* populations (Figure 3). Findings fluctuated within the same regions across the years (Figure 3). Relative CarE activities ranged from 1.00 ± 0.06 (ZJ-2015) to 2.83 ± 0.18 (GA-2013), resulting in a 2.8-fold variation in esterase activity (Figure 3). There were also significant differences in relative glutathione *S*-transferase activities, ranging from 1.00 ± 0.05 (JL-2012) to 2.96 ± 0.13 (GA-2013), with a 3.0-fold variation (Figure 3). The relative cytochrome P450-dependent monooxygenase activity varied from 1.00 ± 0.07 (JL-2012) to 3.36 ± 0.20 (XY-2015), with a 3.4-fold variation (Figure 3).

### Pair-wise correlation analysis

There were no correlations between insecticides (Table 4) and no significant correlations between enzyme activities (GST and P450) and the susceptibility of *C. suppressalis* populations to the insecticides evaluated (Table 4). Noteworthy correlations between enzyme activities, like esterase, and the susceptibility of *C. suppressalis* populations to triazophos were recorded (r = 0.49, *p* = 0.030) (Table 4). There were no meaningful correlations between the activity of CarE and other insecticides (Table 4).

**TABLE 4:** Correlation coefficients between the log LD_50_ values of the tested insecticides in the field populations of *Chilo suppressalis* from 2012-2021 and the enzyme activities.

	Triazophos	Abamectin	Chlorpyrifos	P450	GST	CarE
Triazophos	1					
Abamectin	−0.086 (0.72)	1				
Chlorpyrifos	−0.17 (0.47)	0.32 (0.09)	1			
P450	0.36 (0.12)	0.093 (0.63)	−0.12 (0.53)	1		
GST	−0.24 (0.32)	0.20 (0.32)	0.060 (0.76)	0.078 (0.69)	1	
CarE	0.49 (0.03)^*^	−0.033 (0.87)	−0.13 (0.51)	0.37 (0.05)	0.34 (0.073)	1

^*^Positive correlation between the LD_50_ value of insecticide and enzyme activity at the 95% significance level.

## Discussion

The extensive use of insecticides has resulted in the development of resistance by numerous important pest species. So far, 656 species have built insecticide resistance to 324 compounds, with 21870 cases of resistance having been reported (Sparks and Nauen, 2015; Mota-Sanchez and Wise, 2022). Agricultural productivity has been jeopardized by the widespread resistance to insecticides by crop pests (Roush and Tabashnik, 1990). Therefore, resistance to insecticides by insect pests is one of the most economically damaging circumstances that growers and pest control professionals face. Examining the sensitivity of *C*. *suppressalis* to insecticide each year can establish possible changes in susceptibility and is crucial to monitoring *C*. *suppressalis* resistance levels and banning ineffective insecticides that do not meet pest thresholds.

Triazophos, a broad-spectrum organophosphorus insecticide, was used to manage rice insect pests in China in the early 1990s (Jiang et al., 2001; Rani et al., 2001), becoming the most extensively used insecticide in crop protection against *C*. *suppressalis* in China thanks to its low cost and excellent efficacy. However, after prolonged commercial application for several years, elevated levels of resistance to triazophos were observed in the Zhejing province in 1999 (Jiang et al., 2001). And this resistance to triazophos continued to significantly increase in the years ahead (He et al., 2007; Hu et al., 2010; Zhang et al., 2011; He et al., 2012; He et al., 2013; Su et al., 2014a; Su et al., 2014b). Recent studies established that resistance to triazophos increased to high levels in numerous provinces in China due to extensive usage of the insecticide (Mao et al., 2019; Zhao, 2019). Since then, triazophos has not been recommended for use in the management of *C*. *suppressalis* anymore in the areas where *C*. *suppressalis* had developed high resistance to the insecticide. A comparison of data with those of preceding years revealed that pest insect resistance to triazophos decreased significantly over the years after the suspended application of triazophos (Su et al., 2014a). Additionally, the temporary fluctuations in localized resistance were probably caused by differences in the doses and varieties of insecticide applications, collection time of *C. suppressalis* and rice varieties (Zhao et al., 2021). Investigations in the past have registered low levels of cross-resistance to multiple insecticides, including triazophos, chlorpyrifos, phoxim, isocarbophos, methamidophos, methomyl, abamectin and chlorantraniliprole (Qu et al., 2003; Cao, 2004; Mao et al., 2019). Findings, like triazophos resistance mechanisms in *C*. *suppressalis*, higher esterase activity, and microsomal O-demethylase and AChE insensitivity have also pointed to the possibility of cross-resistance to organophosphorus insecticides (Qu et al., 2003). However, unlike the results in a previous study (Mao et al., 2019), there were no significant correlations between the resistance to triazophos and chlorpyrifos or abamectin in this study, possibly because of the differences in collection sites, insecticide applications, operators, death standards and conditions during the bioassays. Regarding the increased susceptibility due to the suspended application of triazophos, the rotation of insecticides from different modes of action groups provides the best option for minimizing the development of resistance. For instance, rotating triazophos with abamectin could be an effective long-term resistance management strategy.

Chlorpyrifos was recommended as an alternative means of managing *C*. *suppressalis*, brown planthopper and rice leaf folder after the prohibition of high toxic insecticides (Xu et al., 2007). The development of resistance to chlorpyrifos by *C*. *suppressalis* has been rapid, with evolution from susceptibility and low (RR = 0.6−28.6 folds) to moderate resistance (RR = 2.3−78.4 folds) occurring between 2005 and 2011 (He et al., 2008; Su et al., 2014a). Moderate levels of resistance to chlorpyrifos have been noted in many provinces of China (Cheng et al., 2010; He et al., 2013; Su et al., 2014b). The increased resistance to chlorpyrifos in China from 2010-2015 was possibly associated with an escalated use of this insecticide against rice planthopper, striped rice borer and rice leaf folder and the cross-resistance to triazophos (Mao et al., 2019). Cross-resistance suggests that rotating triazophos with chlorpyrifos may not be an effective long-term resistance management strategy (Qu et al., 2003; Cao, 2004). Therefore, alternating with abamectin could slow the development of resistance to chlorpyrifos. However, susceptibility to chlorpyrifos must be monitored carefully to maintain control efficiency and successful resistance management.

Abamectin, an insecticide against arthropod pests, is of economic importance to horticulture and agriculture (Dybas, 1989). A wide range of insect types have developed resistance to abamectin around the world, including *C*. *suppressalis*, *Bactrocera dorsalis*, *Blattella germanica*, *Brontispa longissima*, *Deraeocoris brevis*, *Earias vittella*, *Frankliniella occidentalis*, *Helicoverpa armigera*, *Liriomyza trifolii*, *Metaseiulus occidentalis*, *Panonychus citri*, *Panonychus ulmi*, *Plutella xylostella*, *Spodoptera exigua*, *Spodoptera litura*, *Tetranychus cinnabarinus*, *Tetranychus turkestani*, *Tetranychus urticae*, and *Tuta absoluta* (Mota-Sanchez and Wise, 2022). Abamectin became an alternative to high toxic insecticides in 2006 after showing an excellent management efficacy against *C*. *suppressalis* and *Cnaphalocrocis medinalis* Guene (Gao, 2008; He et al., 2012). *C*. *suppressalis* subsequently slowly built resistance to abamectin from 2001 to 2011 (Su et al., 2014a). One inquiry reported that some field populations of *C*. *suppressalis* in China remained susceptible or displayed only low levels of resistance to abamectin in 2010 and 2011 after 10 more years of use (Su et al., 2014b). However, our analyses revealed that *C*. *suppressalis’* resistance to abamectin in China evolved from low to medium levels from 2010 to 2021. Growers’ use of mixtures of abamectin to regulate *C*. *suppressalis* might not culminate in high resistance to abamectin by *C*. *suppressalis* because blending chemical groups with different MoAs slows down the process of selection for resistance (Su et al., 2014a). While abamectin has been used to manage *C*. *suppressalis* since 1998, it was only recommended as an alternative solution after *C*. *suppressalis* had become highly resistant to trizophos, monosultap and other insecticides (Zhang, 2012). Therefore, scientists must also carefully monitor susceptibility to abamectin to maintain control efficiency and successful resistance management.

Insecticide resistance is probably mediated by the metabolism of insecticides through detoxifying enzymes before they reach their targets (Heckel, 2012; Yang et al., 2020). And many past reports have found an association between insect resistance to insecticides and increases in the activities of these detoxifying enzymes (CarE, GST, and P450) (Zhao et al., 2021). Our investigation established a significant confirmatory correlation between resistance to triazophos and the activity of CarE, suggesting that CarE may be involved in the resistance of these field populations to triazophos. This finding is consistent with a revelation that enhancing the activity of EST (esterase) leads to resistance to triazophos (Qu et al., 2003; Mao et al., 2019). However, our study established no significant correlations between chlorpyrifos or abamectin and enzyme activity.

## Conclusion

Our investigation found that field populations of *C*. *suppressalis* developed moderate to high levels of resistance to triazophos but still showed susceptibility, and low to moderate levels of resistance to chlorpyrifos and abamectin, indicating that insecticide resistance management programs are crucial to regulating pest insects. Hence, triazophos application should be suspended to control *C*. *suppressalis*, while the frequent use of chlorpyrifos and abamectin should be reduced in Central China. The fluctuations in resistance within the same regions across the years and within different regions in the same year could be the result of differences in insecticide applications and rice varieties. Because *C*. *suppressalis* is not a long-distance migratory insect pest, in theory, insecticide application practices and subsequent evolution of resistance to insecticide in one region would not influence the development of resistance in another region. Therefore, the geographical and temporal distribution of insecticide resistance must be scrutinized in detail. Resistance to insecticide by *C*. *suppressalis* should be monitored continuously in Central China. However, resistance monitoring only determines shifts in susceptibility. To avoid resistance, biological control, crop rotation, transgenic plants and cultural practices must be implemented, along with insecticide application strategies, such as alternating the use of insecticides, changing mixtures of insecticides and reducing insecticide application frequencies.

## Data Availability

The original contributions presented in the study are included in the article/Supplementary Material, further inquiries can be directed to the corresponding author.
